# Gene polymorphisms in pattern recognition receptors and susceptibility to idiopathic recurrent vulvovaginal candidiasis

**DOI:** 10.3389/fmicb.2014.00483

**Published:** 2014-09-23

**Authors:** Diana C. Rosentul, Corine E. Delsing, Martin Jaeger, Theo S. Plantinga, Marije Oosting, Irene Costantini, Hanka Venselaar, Leo A. B. Joosten, Jos W. M. van der Meer, Bertrand Dupont, Bart-Jan Kullberg, Jack D. Sobel, Mihai G. Netea

**Affiliations:** ^1^Department of Internal Medicine, Radboud University Medical CenterNijmegen, Netherlands; ^2^Nijmegen Institute for Infection, Inflammation and Immunity (N4i), Radboud University Medical CenterNijmegen, Netherlands; ^3^Centre for Molecular and Biomolecular Informatics, Nijmegen Center for Molecular Life Science, Radboud University Medical CenterNijmegen, Netherlands; ^4^Hôpital Necker-Enfants MaladesParis, France; ^5^Department of Medicine, School of Medicine, Wayne State UniversityDetroit, MI, USA; ^6^Division of Infectious Diseases, Harper University HospitalDetroit, MI, USA

**Keywords:** RVVC, genetic variation, pattern recognition receptors, cytokines

## Abstract

**Objective:** Approximately 5% of women suffer from recurrent vulvovaginal candidiasis (RVVC). It has been hypothesized that genetic factors play an important role in the susceptibility to RVVC. The aim of this study was to assess the effect of genetic variants of genes encoding for pattern recognition receptors (PRRs) on susceptibility to RVVC.

**Study design:** For the study, 119 RVVC patients and 263 healthy controls were recruited. Prevalence of polymorphisms in five PRRs involved in recognition of *Candida* were investigated in patients and controls. *In silico* and functional studies were performed to assess their functional effects.

**Results:** Single nucleotide polymorphisms (SNPs) in *TLR1*, *TLR4*, *CLEC7A*, and *CARD9* did not affect the susceptibility to RVVC. In contrast, a non-synonymous polymorphism in *TLR2* (rs5743704, Pro631His) increased the susceptibility to RVVC almost 3-fold. Furthermore, the *TLR2* rs5743704 SNP had deleterious effects on protein function as assessed by *in silico* analysis, and *in vitro* functional assays suggested that it reduces production of IL-17 and IFNγ upon stimulation of peripheral blood mononuclear cells with *Candida albicans*. No effects were observed on serum mannose-binding lectin concentrations.

**Condensation:** This study demonstrates the association of susceptibility to RVVC with genetic variation in TLR2, most likely caused by decreased induction of mucosal antifungal host defense.

**Conclusion:** Genetic variation in *TLR2* may significantly enhance susceptibility to RVVC by modulating host defense mechanisms against *Candida*. Additional studies are warranted to assess systematically the role of host genetic variation for susceptibility to RVVC.

## INTRODUCTION

*Candida* microorganisms, especially *Candida albicans*, often colonize the genital tract in women, and under certain conditions are responsible for mucosal inflammation (Hurley, 1977; Fleury, 1981; Odds, 1988; Fidel and Sobel, 1996; Giraldo et al., 2000; Sobel, 2007). Vulvovaginal candidiasis (VVC) is a frequent consequence of *Candida* infection, accompanied by variable pruritus, soreness, rash, and vaginal discharge, with patients experiencing a strong discomfort. Most women have at least one event of VVC in their lifetime, while up to 5–8% suffer from recurrent vulvovaginal candidiasis (RVVC), defined as at least three infections per year (Hurley, 1977; Sobel, 2007).

Known risk factors of vulvovaginal candidiasis include diabetes, pregnancy, and therapy with glucocorticosteroids, immunosuppressive drugs, and antibiotics (Milsom and Forssman, 1985; Sobel, 1985; Kent, 1991). However, the vast majority of RVVC patients are healthy women without any identifiable predisposing or episode precipitating factors (Morton, 1977; Sobel, 1985,^1993^; Kent, 1991; Mardh et al., 2002). Moreover, no distinct *C. albicans* strains, the species that causes more that 90% of the VVC episodes, have been described in RVVC patients, arguing against microbiological factors as the major determinants of VVC or susceptibility to recurrent disease (Kent, 1991; Sobel, 1993). Thus, it has been hypothesized that host genetic factors may be a major component determining susceptibility to RVVC.

The innate immune system provides the first barrier against vulvovaginal *Candida* infection. Pattern recognition receptors (PRRs) on innate immune cells sense molecular moieties on the surface of microorganisms, and thereafter induce an intracellular signal that stimulates production of effector molecules such as cytokines or defensins. Two classes of PRRs have been reported to be the main recognition receptors for *C. albicans*: Toll-like receptors (TLRs) and C-type lectin receptors (CLRs; Netea et al., 2008). TLR4 recognizes fungal cell wall mannans, while TLR2/TLR1 and TLR2/TLR6 heterodimers recognize *Candida* phospholipomannan (Jouault et al., 2003). Additionally, TLR2 synergizes with DECTIN-1, the receptor for β-glucan, for the induction of proinflammatory cytokines (Brown et al., 2003; Gantner et al., 2003). DECTIN-1 can also induce TLR-independent signals for the production of IL-17, IL-6, and IL-10 through a Syk/CARD9-dependent pathway (Gow et al., 2007). Single nucleotide polymorphisms (SNPs) in both TLRs and CLRs have been described to be associated with an increased susceptibility to both systemic (Plantinga et al., 2012) and oropharyngeal (Plantinga et al., 2010) candidiasis, and we hypothesized that similar effects may be exerted on the susceptibility to RVVC.

The aim of this study was to assess the impact of the SNPs in the genes coding for DECTIN-1 (Tyr238X, rs16910526), CARD9 (Ser12Asn, rs4077515), TLR1 (Arg80Thr, rs5743611), TLR2 (Pro631His, rs5743704), and TLR4 (Asp299Gly, rs4986790; Thr399Ile, rs4986791) on the susceptibility to RVVC.

## MATERIALS AND METHODS

### ETHICS STATEMENT

The inclusion of patients and controls in this study was approved by the Institutional Review Boards of the Radboud University Medical Centre, School of Medicine, Wayne State University and Hôpital Necker. Enrollment took place between January 2010 and December 2011. Patients gave written informed consent and the study was in accordance to the declaration of Helsinki.

Enrolment of healthy controls for blood donations was approved by the Institutional Review Board of the Radboud University Medical Centre.

### PATIENTS AND CONTROL SUBJECTS

The patient cohort consisting of 119 RVVC patients were recruited at Wayne State University School of Medicine (Detroit, MI, USA), Radboud University Nijmegen Medical Center (Nijmegen, Netherlands) and Hôpital Necker (Paris, France). Patients were enrolled during episodes of acute vaginitis or, if asymptomatic, while receiving maintenance fluconazole therapy. Inclusion criteria for the study were: healthy women above 18 years of age, diagnosed with at least three documented episodes of VVC in a year, microbiologically validated and all caused by *C. albicans*. Exclusion criteria were use of any immunosuppressive therapy (including steroids), diabetes, pregnancy, and HIV infection. EDTA venous blood was collected after obtaining written informed consent. Asymptomatic healthy controls without a history of vaginal *Candida* infections (*N* = 263) were recruited at Radboud University Nijmegen Medical Center, Nijmegen, Netherlands and gave written informed consent. All patients and controls were of Western-European genetic background.

### GENOTYPING OF THE SINGLE NUCLEOTIDE POLYMORPHISMS

Genomic DNA was isolated from whole blood using the Qiagen (Valencia, CA, USA) isolation kit and following the standard protocol. The genotype for the *CLEC7A* (*DECTIN-1*) Tyr328X (rs16910526) and *CARD9* Ser12Asn (rs4077515) polymorphisms in the patients was screened by the TaqMan SNP assay C_33748481_10 and C_25956930_20, respectively, (Applied Biosystems, Foster City, CA, USA). The genotype for the *TLR1* polymorphism Arg80Thr (rs5743611) was assessed with the TaqMan SNP assay C_27855269_10. The genotype of *TLR2* Pro631His (rs5743704) was assessed by applying a predesigned TaqMan SNP assay C_25607736_10. The genotyping for the *TLR4* polymorphisms Asp299Gly (rs4986790) and Thr399Ile (rs4986791) was performed with the TaqMan SNP assay C_11722238_20 and C_11722237_20, respectively. The TaqMan qPCR assays were performed on the 7300 ABI Real-Time polymerase chain reaction system (Applied Biosystems). Positive and negative controls were included in the assays.

### BIOINFORMATIC ANALYSIS FOR THE TLR2 POLYMORPHISM rs5743704

The *TLR2* protein information was obtained from Swissprot accession code O60603, and OMIM accession code 603028, using the MRS server^1^. We used the PROSITE server^2^ to retrieve information about the TLR2 protein domains. No protein structure is available for the complete TLR2 protein at the moment. However, the cytoplasmic TIR domain is known and can be found as PDB-file 1O77 (Tao et al., 2002). This structure in this file contains a mutation on position 713 and misses one loop, but the remaining residues are 100% identical to the sequence of TLR2.

The extracellular domain up to residue 509 is 100% identical to the protein in PDB file 2Z7X (Jin et al., 2007). The Pro631His polymorphism is located in the region linking the TIR domain with the intracellular domain. We used the automatic modeling script in the YASARA & WHAT IF Twinset (Krieger et al., 2002) to extend the known structures and to model missing loops in these structures. As a result, we produced models of the N- and of the C-terminal domain, which are both accurate, because they are based on the known structure of that protein. These structures are extended with residues of the linking region. Additionally, we used HOPE^3^, a next generation bioinformatics web server that performs automatic mutant analysis (Venselaar et al., 2010). Besides this, an analysis of the pathogenicity of the Pro631His polymorphism in *TLR2* was performed using conservation and structural information. The Polyphen-2, SIFT, PANTHER, snps3D, and the SNAP servers where employed for this analysis.

### CYTOKINE STIMULATION ASSAYS

Peripheral blood mononuclear cells (PBMCs) were isolated from 73 healthy volunteers by Ficoll-Paque gradient. All healthy volunteers gave written informed consent. 0.5 × 10^6^ PBMCs/well were plated in round bottom wells plates. Subsequently, incubation with *C. albicans* blastoconidia (1 × 10^6^/mL, heat-killed at 100^∘^C for 1 h) was performed for 24 h, 48 h or 7 days. The incubation time varied for each cytokine: 24 h for IL-1β and IL-6, 48 h for IFNγ and IL-10, and 7 days for IL-17 (with the addition of 10% human serum). After the incubation time, cytokines were measured in the supernatants by ELISA (R&D Systems, MN, USA or Sanquin Research, Amsterdam, Netherlands). Detection limits were 40 pg/mL, except for the IFNγ ELISA (12 pg/mL). Mannose-binding lectin (MBL) concentrations in serum were measured by ELISA (Bioporto, Gentofte, Denmark).

### STATISTICAL ANALYSIS

For the analysis of the polymorphisms in the PRRs, statistical comparisons of frequencies were made between RVVC versus control subjects. We used SPSS 16.0 software to perform Chi-square tests and the 5% Confidence Intervals Odds ratio to calculate the risk. Furthermore, for the analysis of the effect of the *TLR2* genotype on the *in vitro* cytokine production, a non-parametrical Kruskal–Wallis analysis was performed, and for the circulatory MBL measurement we used the Mann–Whitney *U*-test. Both analyses were done using GraphPad Prism version 4.00 for Windows, GraphPad Software, San Diego, CA, USA.

## RESULTS

### THE INFLUENCE OF POLYMORPHISMS IN PRR GENES ON THE SUSCEPTIBILITY TO RVVC

As a polymorphism in the gene encoding the CLR DECTIN-1 (*CLEC7A*) has previously been shown to be associated with an increased susceptibility to fungal infections, a first set of comparisons assessed the prevalence of *CLEC7A* and *CARD9* polymorphisms in control individuals and patients with RVVC (**Table 1**). No effects of *CLEC7A* (Tyr238X, rs16910526) and *CARD9* (Ser12Asn, rs4077515) polymorphisms on the susceptibility to RVVC have been observed. In a second set of experiments, the prevalence of polymorphisms in the genes coding for TLR1 (Arg80Thr, rs5743611), TLR2 (Pro631His, rs5743704), and TLR4 (Asp299Gly, rs4986790; Thr399Ile, rs4986791) was also assessed. These analyses revealed that the polymorphisms in the *TLR1* and *TLR4* genes did not affect the susceptibility to RVVC. In contrast, the genotype for the Pro631His polymorphism on the *TLR2* gene was associated with a 2.705-fold increase (*P*-value 0.046) in susceptibility to RVVC in the patient cohort. All genetic distributions are in Hardy–Weinberg equilibrium (**Table 1**).

**Table 1:** Genetic association of recurrent vulvovaginal candidiasis (RVVC) with polymorphisms in DECTIN-1, CARD9, TLR1, TLR2, and TLR4 genes.

Polymorphism	Group	WT	HET	HOM	In HWE (yes/no)	P-value	OR (95% CI)
DECTIN-1 Tyr238Stop	Controls (*N* = 263)	219 (83.3%)	44 (16.7%)	0 (0%)	Yes	0.852	0.95 (0.53-1.70)
	RVVC (*N* = 119)	100 (84.0%)	19 (16.0%)	0 (0%)	Yes		
CARD9Ser12Asn	Controls (*N* = 263)	90 (34.2%)	127 (48.3%)	46 (17.5%)	Yes	0.563	1.03 (0.76-1.40)
	RVVC (*N* = 119)	43 (36.1%)	51 (42.9%)	25 (21.0%)	Yes		
TLR1Arg80Thr	Controls (*N* = 263)	232 (88.2%)	29 (11.0%)	2 (0.8%)	Yes	0.46	1.41 (0.81-2.43)
	RVVC (*N* = 119)	100 (84.0%)	17 (14.3%)	2 (1.7%)	Yes		
**TLR2Pro631His**	**Controls (*N* = 263**)	**252 (95.8%)**	**11 (4.2%)**	**0 (0%)**	**Yes**	**0.046**	**2.71 (1.15-5.85)**
	**RVVC (*N* = 119)**	**107 (89.9%)**	**11 (9.2%)**)	**1 (0.9%)**	**Yes**		
TLR4Asp299Gly	Controls (*N* = 263)	230 (87.5%)	32 (12.2%)	1 (0.5%)	Yes	0.791	0.90 (0.47-1.73)
	RVVC (*N* = 119)	105 (88.2%)	14 (11.8%)	0 (%)	Yes		
TLR4Thr399Ile	Controls (*N* = 263)	230 (87.5%)	32 (12.2%)	1 (0.3%)	Yes	0.791	0.90 (0.47-1.73)
	RVVC (*N* = 119)	105 (88.2%)	14 (11.8%)	0 (%)	Yes		

*Data are presented as number of individuals (% major or minor allele), *P*-value and OR (95% CI).*

Bold indicates statistically significant association.

^*^Chi-square (χ^2^) test. WT, wild-type; HET, heterozygous; HOM, homozygous; HWE, Hardy-Weinberg equilibrium.

### BIO-INFORMATIC ANALYSIS TLR2 rs5743704 POLYMORPHISM

Because the *TLR2* Pro631His polymorphism was associated with an increased susceptibility to RVVC, the functional effects of this genetic variation were studied. In a first analysis, an *in silico* evaluation of the effect of the Pro631His polymorphism on the TLR2 protein was performed. We evaluated its effect at the genomic level, by performing multiple sequence alignment of the homologous DNA sequences from 35 other species via the Ensembl server. The cytosine base is conserved at the chromosome 4 nucleotide position 154625451, being also the ancestral allele, evolutionary conservation that underlines a high likelihood that this position in the molecule is functionally important.

**FIGURE 1:**
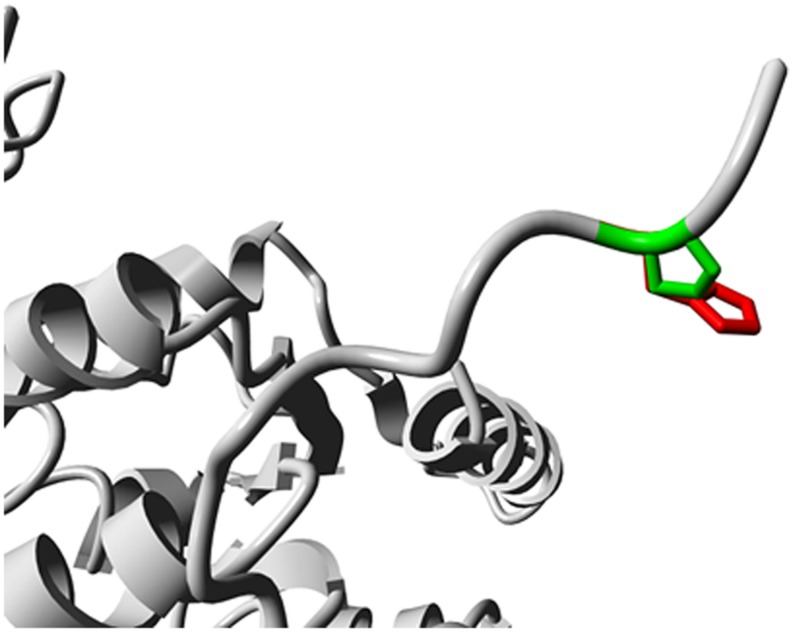
**Screenshot of the 3D homology model of the TLR2 protein using the YASARA software.** The side chains of the wild-type and mutant residues are shown in green and red, respectively.

To analyze the effects of the SNP rs5743704 on the TLR2 protein, we analyzed the possible domains identified via the Prosite server. The amino acid change for the Pro631His polymorphism affects an amino acid residue that is located near the TIR domain (residue 639–784). To visualize the Pro631His polymorphism on the 3D structure of the TIR domain, we evaluated the homology model using the YASARA software. A screenshot of the 3D homology model is shown in **Figure 1**. The amino acid change is situated between the intracellular domain and the TIR domain, and might introduce a loss in rigidity when substituting proline by histidine.

Finally, the HOPE server predicted that due to the fact that the polymorphism introduces an amino acid with different characteristics, this would affect the function of the molecule. The wild-type residue proline is rigid, resulting in a characteristic backbone conformation that might be required at this position. Additionally, proline is more hydrophobic than histidine, and this may also influence the function of the molecule through loss of hydrophobic interactions on the surface of the protein. This hypothesis was also tested through a series of web servers to predict the pathogenicity of the Pro631His amino acid change. As shown in Supplementary Table 1, according to the Polyphen-2, SIFT, PANTHER, and snps3D, the effect of the Pro631His polymorphism is predicted detrimental for TLR2 function.

### THE EFFECT OF THE TLR2 Pro631His POLYMORPHISM ON THE PRODUCTION OF INFLAMMATORY MEDIATORS

**FIGURE 2:**
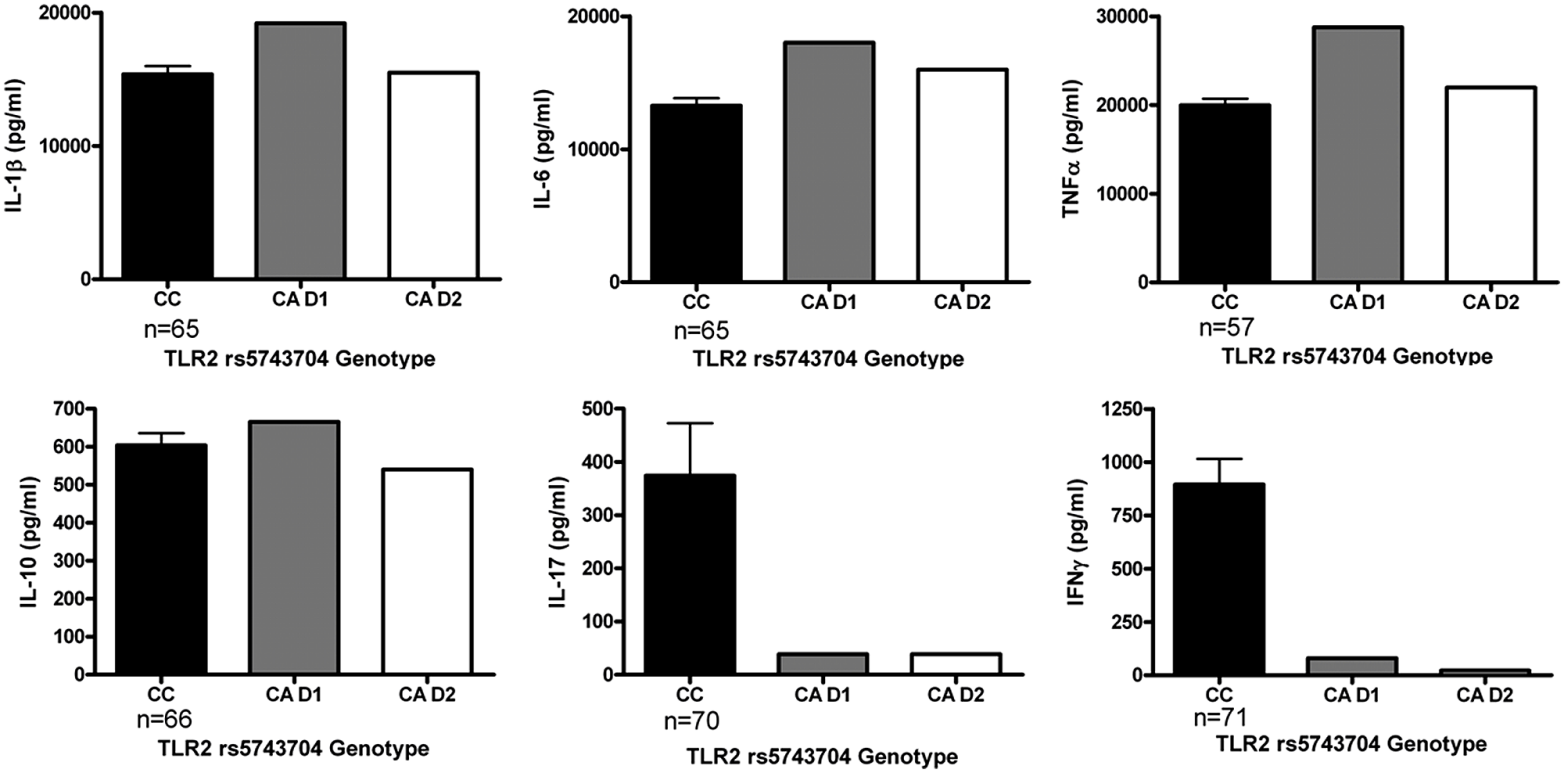
**Functional analysis on the stimulation with heat-killed *Candida albicans* of peripheral blood mononuclear cells (PBMCs) with different TLR2 Pro631His genotypes.** Cytokine production capacity of TNFα, IL-1β, IL-6, IL-10, IL-17, and IFNγ was compared between the cells obtained from healthy volunteers bearing the wild-type or the variant allele of the *TLR2* polymorphism. D1: Donor 1, D2: Donor 2. Data are presented as means ± SEM.

**FIGURE 3:**
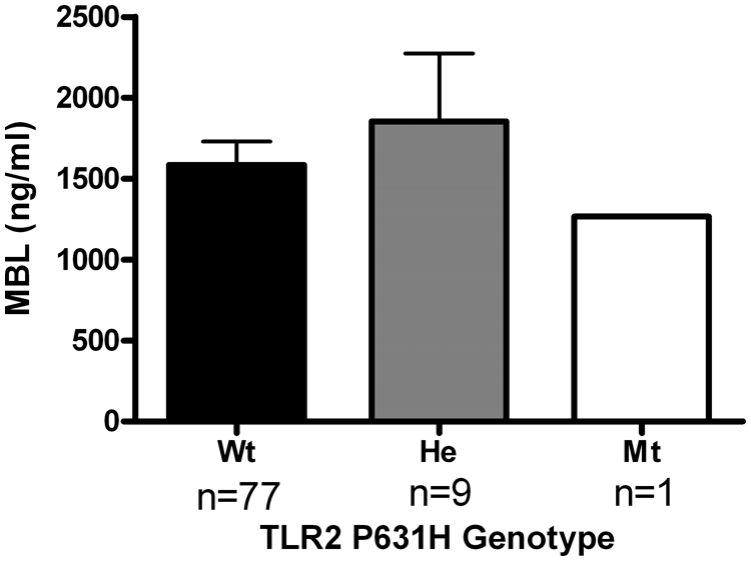
**Analysis on the mannose-binding lectin (MBL) concentrations in serum obtained from RVVC patients bearing the wild-type or the variant allele of the *TLR2* polymorphism Pro631His.** Wt, wild-type; He, heterozygous. Data are presented as means ± SEM.

In order to assess the functional consequences of the *TLR2* Pro631His polymorphism in further detail, cytokine production of PBMCs stimulated with heat-killed *C. albicans* was assessed in a group of healthy volunteers. The major limitation of this part of the study was that only two individuals in this group were heterozygous for the variant allele, which precludes drawing definitive conclusions. However, we observed that whereas the IL-1β, IL-6, and TNFα production was in the same range comparing the different genotype groups, both individuals bearing the mutated allele displayed a strongly diminished release of the T-cell derived cytokines IFNγ and IL-17 (**Figure 2**), known to be important for antifungal host defense (Pandiyan et al., 2011; van de Veerdonk et al., 2011; Johnson et al., 2012). In contrast, MBL circulating concentrations in the RVVC patients did not differ depending on the *TLR2* genotype (**Figure 3**).

## DISCUSSION

Vulvovaginal candidiasis is one of the most common infections in women. Despite known risk factors such as the hormonal status, diabetes, pregnancy or immunosuppressive therapy (Milsom and Forssman, 1985; Sobel, 1985,^1993^; Kent, 1991; Nawrot et al., 2000), the majority of the patients are healthy women who are not exposed to these conditions. In the present study, we explored whether genetic variation in genes coding for PRRs involved in *Candida* recognition (Jouault et al., 2003) affects susceptibility to RVVC. We show that a non-synonymous polymorphism in *TLR2* is associated with increased susceptibility to RVVC. *In silico* assessment suggested functional consequences of the *TLR2* polymorphism, and this hypothesis is supported by the decreased production of T-cell derived cytokines IFNγ and IL-17 in two individuals bearing the mutation. However, a definitive conclusion was not able to be drawn due to the low number of volunteers with *TLR2* SNP identified in the functional studies.

Genetic variants in PRRs have been previously reported to influence predisposition to RVVC. This has been described in a family of patients with a complete deficiency of DECTIN-1, due to an early stop codon mutation in the *CLEC7A* gene coding for the DECTIN-1 receptor (Ferwerda et al., 2009). Functional consequences of DECTIN-1 deficiency were demonstrated to include impaired induction of both innate and adaptive Th17 immune responses. Furthermore, several other studies have shown the involvement of adaptive immunity in RVVC in humans (Babula et al., 2005; De Luca et al., 2013). In addition to RVVC occurrence in two of the three patients with DECTIN-1 deficiencies, all suffered from onychomycosis. This mutation appears to be relatively common, up to 15% heterozygosity in populations of European origin, allowing epidemiological studies in heterozygous patients (Ferwerda et al., 2009). Moreover, heterozygosity in the *CLEC7A* gene is associated with increased mucosal *Candida* colonization (Plantinga et al., 2009). Thus, we assessed its impact in the present study. The loss of one functional allele, however, does not seem to have a major impact on the susceptibility to RVVC. This implies that only a complete loss-of-function in the molecule is associated with a significant risk for infection. In addition, it has also recently been shown that this polymorphism does not affect the predisposition to disseminated *Candida* infections (Plantinga et al., 2009; Rosentul et al., 2011).

CARD9 is an adaptor molecule mediating signals induced by DECTIN-1, but also other CLRs. Its role in antifungal immunity has been demonstrated both in knock-out mouse studies (Gross et al., 2006), as well as by the increased susceptibility to severe fungal infections in patients defective in this molecule due to an early stop codon in position Gln295Stop (Glocker et al., 2009). However, this mutation is rare and has not been reported in other individuals. The evaluation of the effects of a known *CARD9* Ser12Asn polymorphism did not yield a significant association with RVVC susceptibility in the present study. Similarly, we have previously shown that this polymorphism does not influence the susceptibility to candidemia (Rosentul et al., 2011).

In addition to CLRs, TLRs are the second major family of PRRs involved in the recognition of fungi in general, and *Candida* in particular, TLR2, TLR4, and TLR1 being most important in antifungal immunity (Netea and Marodi, 2010). While *TLR1* polymorphisms have been recently shown to influence susceptibility to candidemia (Plantinga et al., 2012), no such effect was apparent in patients with RVVC. Similarly, two *TLR4* polymorphisms that have been previously associated with susceptibility to Gram-negative sepsis and other infections did not have a significant effect on susceptibility to RVVC (Schroder and Schumann, 2005).

In contrast, the *TLR2* Pro631His polymorphism induced an almost threefold increase in the susceptibility to RVVC. TLR2 is an important PRR for *C. albicans* recognition, activating innate immune responses both alone and in synergy with DECTIN-1 (Ferwerda et al., 2008). The deficiency of TLR2 influences susceptibility to systemic candidiasis in mice (Netea et al., 2004), but no studies have been performed in vaginal candidiasis models. The *in silico* analysis using homology modeling and conservation analysis suggests detrimental effects of the mutation on the function of the receptor. This is supported by the study of Etokebe et al. (2010) that the Pro631His polymorphism has a dominant negative effect on the TLR2 signaling in HEK-293T cells. Finally, we studied the functional relevance of this polymorphism in primary cells from individuals bearing the various alleles. Although we were able to assess cytokine production in only two individuals with a mutant *TLR2* allele, both of them consistently produced very low amounts of the T-cell derived cytokines IFNγ and IL-17, mediators that are crucial for mucosal antifungal defense (Ferwerda et al., 2009; van de Veerdonk et al., 2011). This observation is supported by the finding of Ben-Ali et al. (2011) who demonstrated that the 631His *TLR2* variant leads to reduced NF-κB activation.

In addition to the effects of the *TLR2* polymorphism on cytokine production, we have also assessed its influence on the concentrations of MBL. MBL is a circulating receptor for mannose residues which activates the complement system and opsonizes *Candida* (Babula et al., 2005). MBL deficiency is associated with an increased susceptibility to RVVC (Babula et al., 2003; Kaoro Horie Wojitani et al., 2012). However, no differences in serum MBL concentrations were found between individuals bearing different *TLR2* alleles, showing that the effect of the *TLR2* Pro631His polymorphism on the RVVC susceptibility is independent of the production of MBL. Ideally, the cytokine analyses should be extended to assessment in lavage fluids or cells. However, these materials were not collected from the patients presented in this manuscript, and future studies should address this aspect.

In conclusion, in this study we provide evidence that polymorphisms in PRRs may play an important role in susceptibility to RVVC in otherwise healthy women. While *CLEC7A*, *CARD9*, *TLR1*, and *TLR4* polymorphisms had no impact, the *TLR2* Pro631His polymorphism was associated with an almost 3-fold increase in susceptibility to RVVC. These data demonstrate the role of TLR2 genetic variation in innate immunity genes for RVVC, and future investigations are warranted in larger cohorts if possible to replicate and extend the results of the present study.

## SUPPLEMENTARY MATERIAL

The Supplementary Material for this article can be found online at http://www.frontiersin.org/journal/10.3389/fmicb.2014.00483/abstract

## Conflict of Interest Statement

The authors declare that the research was conducted in the absence of any commercial or financial relationships that could be construed as a potential conflict of interest.

## Footnotes

1 http://mrs.cmbi.ru.nl/mrs-web

2 http://expasy.org/prosite

3 http://www.cmbi.ru.nl/hope
