# Supplementary material for: Gene polymorphisms in pattern recognition receptors and susceptibility to idiopathic recurrent vulvovaginal candidiasis
Source: Front Microbiol. 2014 Sep 23;5:483. doi: 10.3389/fmicb.2014.00483 (PMC4172055; doi:10.3389/fmicb.2014.00483)
Supplement: Supplementary file 1 [file Table_1.DOCX]

Supplementary Table 1. Predictions on pathogenicity of the Pro631His polymorphism in the *TLR2* gene using different web servers.

| Method | URL | Prediction | Reference |
| --- | --- | --- | --- |
| Polyphen-2 | http://genetics.bwh.harvard.edu/pph2/ | Probably damaging | Adzhubei et al., 2010 |
| SIFT | http://blocks.fhcrc.org/sift/SIFT.html | Non-tolerated | Ng and Henikoff, 2003 |
| PANTHER | http://www.pantherdb.org/tools/csnpScore.do | Deleterious | Thomas et al., 2003 |
| snps3D | http://www.snps3d.org/ | Deleterious | Yue et al., 2006 |
| SNAP | http://cubic.bioc.columbia.edu/services/SNAP/ | Non-neutral | Bromberg and Rost, 2007 |
